# Olive oil-derived endocannabinoid-like mediators inhibit palatable food-induced reward and obesity

**DOI:** 10.1038/s42003-023-05295-y

**Published:** 2023-09-21

**Authors:** Nicola Forte, Charlène Roussel, Brenda Marfella, Anna Lauritano, Rosaria Villano, Elvira De Leonibus, Emanuela Salviati, Tina Khalilzadehsabet, Giada Giorgini, Cristoforo Silvestri, Fabiana Piscitelli, Maria Pina Mollica, Vincenzo Di Marzo, Luigia Cristino

**Affiliations:** 1 Institute of Biomolecular Chemistry, National Research Council of Italy, Via Campi Flegrei 34, 80078 Pozzuoli (NA), Italy; 2 Heart and Lung Research Institute of Université Laval, Québec City, QC Canada; 3 Institute for Nutrition and Functional Foods, Centre NUTRISS, Université Laval, Québec City, QC Canada; 4 Department of Biology, University of Naples Federico II, 80126 Naples, Italy; 5 Telethon Institute of Genetics and Medicine, Pozzuoli, Naples, Italy; 6 Institute of Biochemistry and Cell Biology, Consiglio Nazionale delle Ricerche (CNR), Monterotondo Scalo, Rome, Italy; 7 Department of Pharmacy, University of Salerno, Fisciano (SA), Italy; 8 Centro Servizi Metrologici e Tecnologici Avanzati (CeSMA), Complesso Universitario di Monte Sant’Angelo, 80126 Naples, Italy; 9 Task Force on Microbiome Studies, University of Naples Federico II, 80138 Naples, Italy; 10 Canada Excellence Research Chair on the Microbiome-Endocannabinoidome Axis in Metabolic Health, Université Laval, Québec City, QC 61V0AG Canada

**Keywords:** Obesity, Physiology, Risk factors

## Abstract

*N*-oleoylglycine (OlGly), a lipid derived from the basic component of olive oil, oleic acid, and *N*-oleoylalanine (OlAla) are endocannabinoid-like mediators. We report that OlGly and OlAla, by activating the peroxisome proliferator-activated receptor alpha (PPARα), reduce the rewarding properties of a highly palatable food, dopamine neuron firing in the ventral tegmental area, and the obesogenic effect of a high-fat diet rich in lard (HFD-L). An isocaloric olive oil HFD (HFD-O) reduced body weight gain compared to the HFD-L, in a manner reversed by PPARα antagonism, and enhanced brain and intestinal OlGly levels and gut microbial diversity. OlGly or OlAla treatment of HFD-L mice resulted in gut microbiota taxonomic changes partly similar to those induced by HFD-O. We suggest that OlGly and OlAla control body weight by counteracting highly palatable food overconsumption, and possibly rebalancing the gut microbiota, and provide a potential new mechanism of action for the obeso-preventive effects of olive oil-rich diets.

## Abstract (web summary)

In mice, lipoamino acids activate the peroxisome proliferator-activated receptor alpha, thus reducing the rewarding properties of palatable food, dopamine neuron firing and the obesogenic effect of a high-fat diet.

## Introduction

The daily consumption of Western diets, consisting of highly palatable and caloric food, represents the main environmental cue triggering addictive-like behaviors and obesity^1,2^. Highly palatable food (HPF) stimulates dopaminergic (DA) neurons in the VTA, thereby activating the reward circuit^3,4^. Short-term exposure to HPF induces long-lasting synaptic plasticity in mesolimbic DA neurons^5^.

Lipoaminoacids, such as OlGly, are endogenous endocannabinoid-like mediators belonging to the large family of the endocannabinoidome (eCBome)^6^. OlGly is endogenously synthesized from oleic acid^7^, the main monounsaturated long-chain fatty acid present in olive oil, a basic component of the Mediterranean diet. Since OlGly undergoes fast degradation by endogenous amidases, a more stable analogue, OlAla, was synthesized and tested, and found to be more efficacious than OlGly in vivo (see below)^8^. Interestingly, evidence exists for the presence also of endogenous OlAla in the brain^9^.

Indeed, OlGly and, particularly, OlAla reverse abuse-related effects of naloxone-precipitated withdrawal behaviors from chronic opiates in mice, while they do not modify tolerance to nociception, hyperthermia, and suppression of activity produced by morphine^8–10^. Recently it was demonstrated that both OlGly and OlAla reduce alcohol intake and preference^11^. More importantly for the purpose of this study, OlGly reduces nicotine preference and withdrawal in mice^12^ by activating PPARα^12^, and GW6471 (GW) (2 mg/kg), a selective PPARα antagonist, prevents OlGly-induced inhibition of nicotine conditioned place preference (CPP)^12^. It was previously demonstrated that PPARα activation reduces VTA DA output^13,14^.

Results from studies in both rodents and humans have suggested that the gut microbiome is modulated by the diet and intervenes in increased consumption of, and dependence from, HPF^15,16^. Indeed, the gut microbiome is increasingly being proposed to play a role in obesity and its related disturbances, such as food addictive behaviors, and to interact with eCBome signaling^17–19^ in both the brain and gut^20,21^. Nevertheless, the involvement in behavioral signs of food choice or preference in rodents of the gut microbiome, and the effects thereupon of eCBome mediators, such as OlGly and OlAla, have not yet been fully elucidated.

The current study aimed at assessing the role of OlGly and OlAla as endogenous, and possibly olive oil-derived, molecules counteracting food-related addictive behaviors and, consequently, high fat-induced increases of food intake and weight gain in mice.

## Results

### OlGly and OlAla do not alter food intake but counteract HPF-induced CPP via PPARα receptor activation

**Fig. 1:**
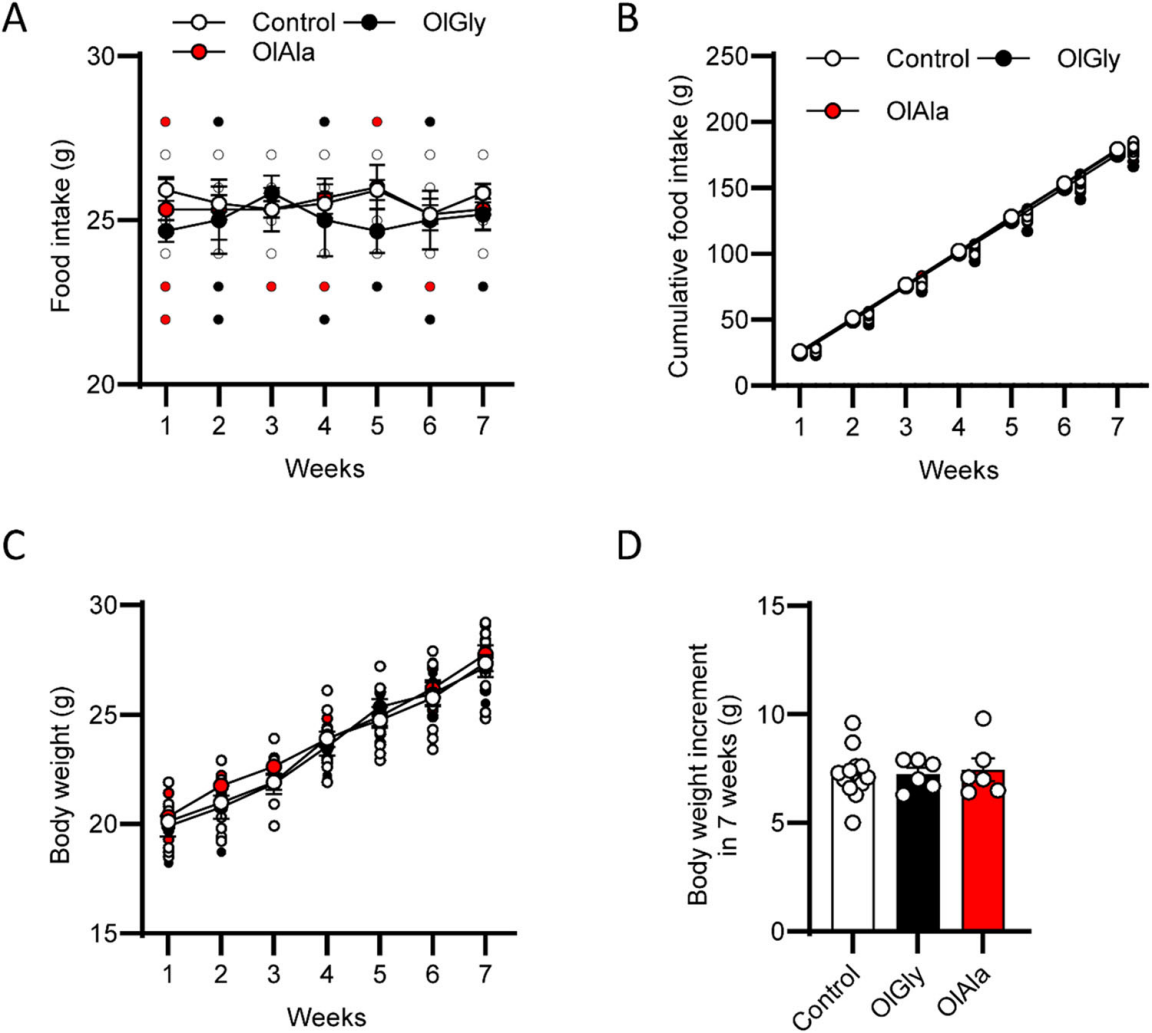
OlGly and OlAla do not alter food intake and body weight by chronic treatment in mice under a standard chow diet. **A** Effect OlGly (50 mg/Kg) or OlAla (50 mg/Kg), on food intake of mice fed with chow. **B** Cumulative food intake (g). **C** Body weight changes following OlGly (50 mg/Kg) or OlAla (50 mg/Kg) administration. **D** Effect of OlGLy or OlAla on the the weight of mice after 7 weeks of treatment. *n* = 12 vehicle, *n* = 6 OlGly, *n* = 6 OlAla.

Before testing whether OlGly and OlAla are good candidates to counteract food craving induced by HPF, we first assessed the effect of their chronic treatment (7 weeks, 3 times per week; 50 mg/kg) on food (chow) intake and body weight in mice fed with a standard mouse diet. Neither parameter was affected by the treatments (Fig. 1A–D).

**Fig. 2:**
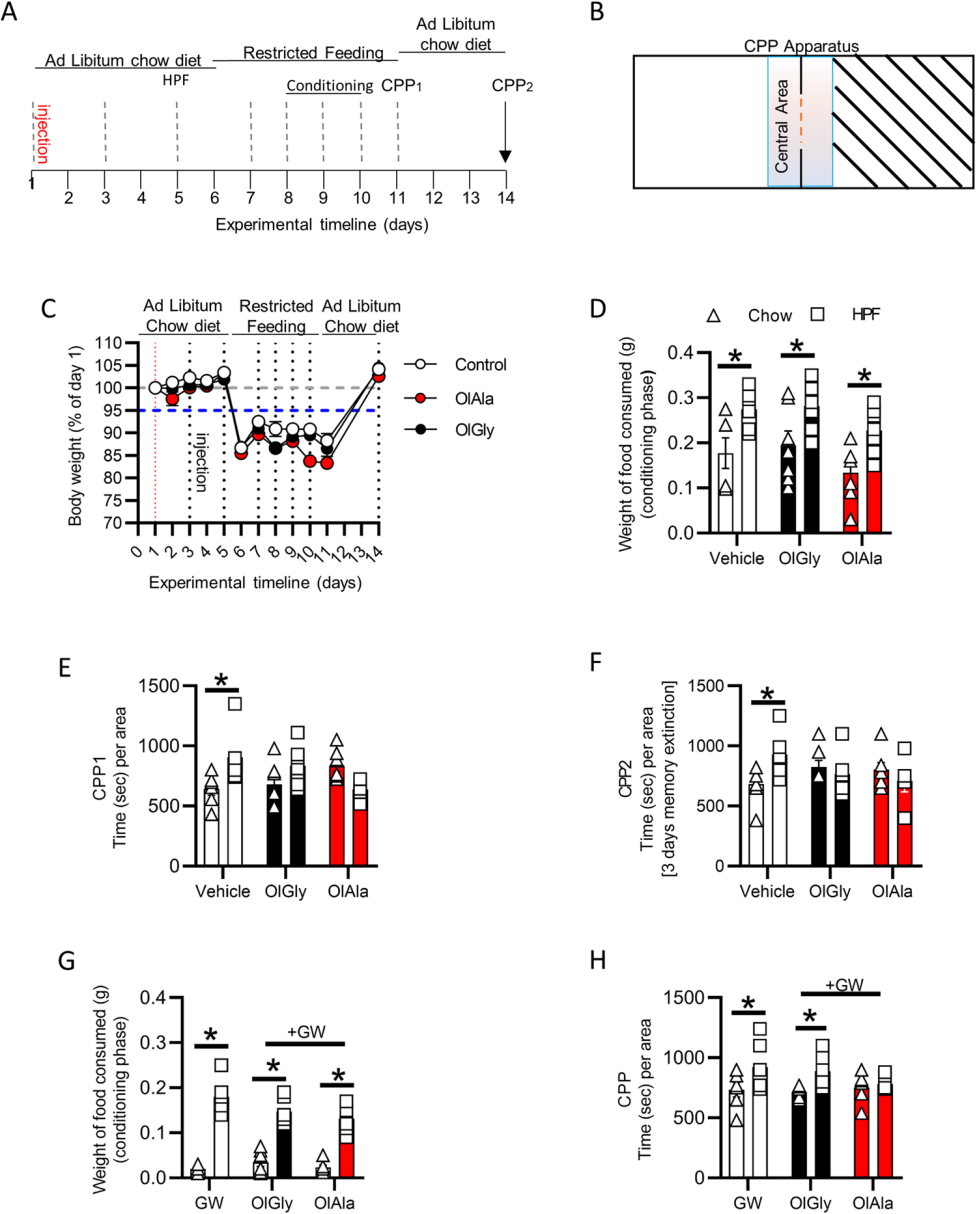
OlGly and OlAla inhibit CPP induced by HPF in a manner prevented by GW in OlGly-treated mice. **A** CPP experimental timeline. **B** Schematic representation of the CPP apparatus. **C** Body weight changes during the CPP experiment in vehicle-, OlGly- (50 mg/Kg) or OlAla-injected (50 mg/Kg) mice. **D** Weight of food consumed (chow food or HPF) per mouse during the conditioning session of vehicle- (control), OlGly- (50 mg/Kg) or OlAla-injected (50 mg/Kg) mice. **E** CPP_1_ test was performed after the conditioning session. **F** CPP_2_ test performed after two days of ad libitum feeding. **G** Weight of food consumed (chow food or HPF) per mouse during the conditioning session in GW (2 mg/Kg), GW (2 mg/Kg) + OlGly (50 mg/Kg) and GW (2 mg/Kg) + OlAla (50 mg/Kg) group. **H** CPP test performed after a conditioning session. *p < 0.05. Two-way ANOVA and Sidak’s multiple comparison test. *n* = 6 GW, 7 GW + OlGly and 7 GW + OlAla mice per group *p < 0.05. Two-way ANOVA and Sidak’s multiple comparison test. *n* = 6 vehicle-, *n* = 7 OlGly-, *n* = 7 OlAla-, *n* = 6 GW-, *n* = 7 GW + OlGly-, *n* = 7 GW + OlAla-injected mice.

Since it was shown that OlGly counteracts nicotine-induced CPP^12^, we tested if this endogenous mediator also affected the rewarding properties of HPF using the apparatus and protocol depicted in Fig. 2A. The results show that mice ate significantly more HPF than standard food regardless of treatment during the conditioning phase (Fig. 2A, B, D). However, during the place preference test phase (when no food was present), mice injected with vehicle spent significantly more time in the room where the HPF had been present, thus confirming the rewarding properties of HPF; in contrast the OlGly- (50 mg/Kg) or OlAla-treated (50 mg/Kg) group spent the same amount of time in both rooms (Fig. 2E).

Short consumption of HPF leads to long-lasting changes in food approach, even when alternated with a standard diet^5^. To test whether our protocol was able to induce these long-lasting effects on food preference (Fig. 2A), conditioned mice were kept for three further days under an *ad libitum* chow diet regimen (Fig. 2C) without pharmacological treatment. Subsequently, CPP was re-tested (CPP2). The results show the persistence of a CPP in mice, which confirmed their preference for the HPF-associated room despite not being under food deprivation. Also this long-lasting effect was prevented by OlGly or OlAla (Fig. 2F).

These data suggest that mice prefer HPF compared to chow and that OlGly or OlAla do not influence either the food choice or the amount of food consumed, indicating that the two drugs do not modify the hedonic value of food or the motivational drive of the animals. However, both compounds were able to counteract the reward-associative properties of HPF.

Our results point the attention to the capability of the OlGly and OlAla to counteract the “addictive” effect of HPF, recapitulating what had been demonstrated using nicotine^12^. It was also previously demonstrated that OlGly and OlAla activate PPARα^8,12^ and that the selective PPARα antagonist GW6471 (GW) (2 mg/kg) prevents OlGly-induced inhibition of nicotine CPP^12^. To test whether the effects described above were also mediated by PPARα, we coadministered either compound with GW (2 mg/kg). GW-treated mice, without and with OlGly or OlAla, still consumed significantly more HPF than chow (Fig. 2G). Furthermore, GW-treated mice still showed CPP for the HFP compartment. While GW was unable to significantly counteract the ability of OlAla to inhibit the rewarding properties of HPF in the CPP test, it did inhibit this effect of OlGly (Fig. 2H).

### OlGly and OlAla decrease HFD-L induced weight gain

**Fig. 3:**
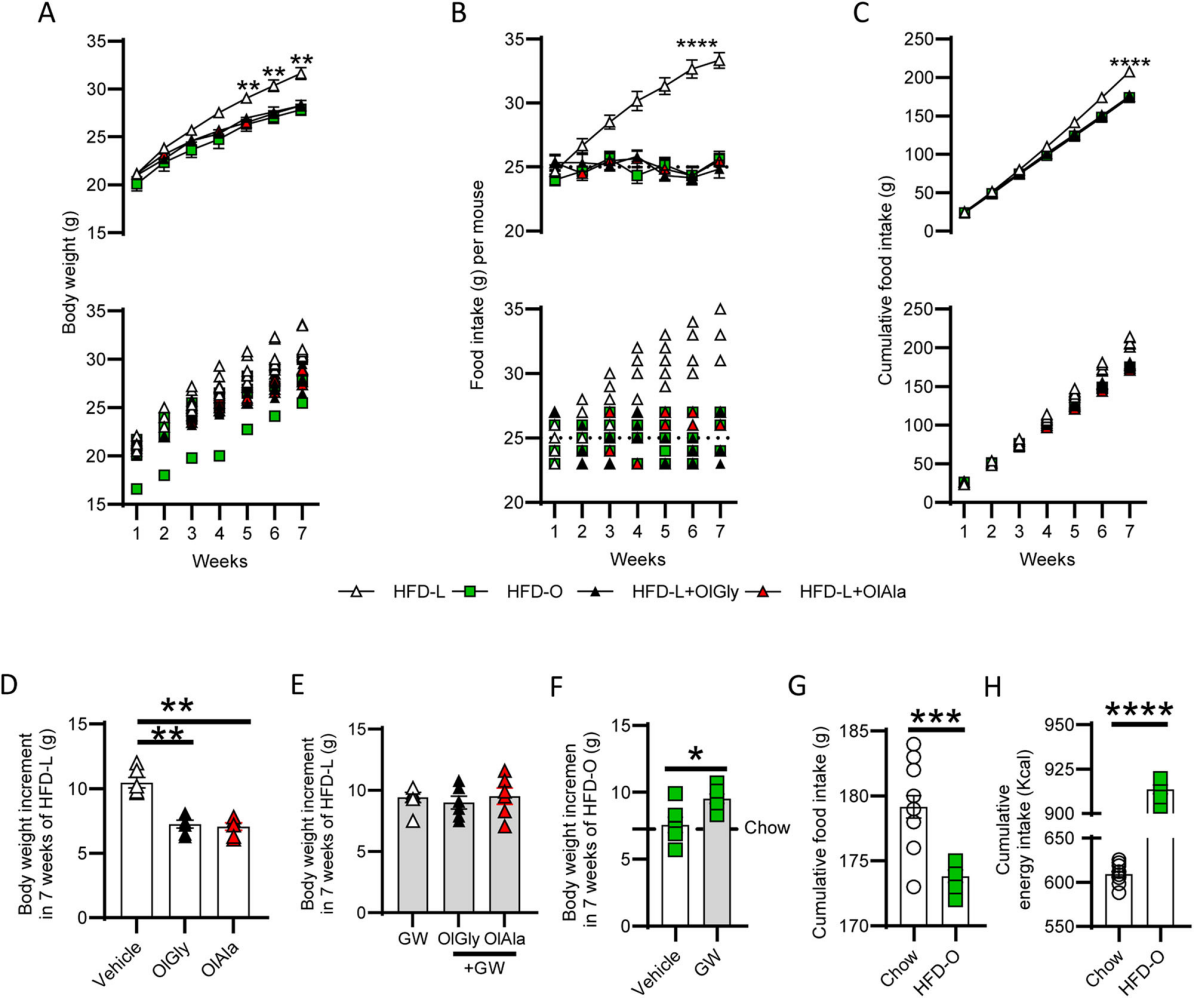
OlGly and OlAla counteract the obesogenic effect of a HFD diet rich in lard. **A** Body weight changes following 7 weeks of HFD-L or HFD-O, and following OlGly (50 mg/Kg) or OlAla (50 mg/Kg) treatment of HFD-L mice.**p < 0.01, one-way ANOVA and Bonferroni post hoc, the graphs show the mean + SEM (upper graph) and the individual data points (lower graph). **B** Effect of OlGly (50 mg/Kg) or OlAla (50 mg/Kg) treatment on mice fed with HFD-L and effect of HFD-O on food intake per week. ****p < 0.0001, one-way ANOVA and Bonferroni post hoc, the graphs show the mean + SEM (upper graph) and the individual data points (lower graph). **C** Effect of OlGly (50 mg/Kg) or OlAla (50 mg/Kg) treatment on mice fed with HFD-L and effect of HFD-O on cumulative food intake. ****p < 0.0001, one-way ANOVA and Bonferroni post hoc, the graphs show the mean + SEM (upper graph) and the individual data points (lower graph). **D** Weight increment after 7 weeks of HFD-L in the vehicle, OlGly and OlAla treated mice; *n* = 6 mice per group, **p < 0.01, one-way ANOVA and Bonferroni post hoc. **E** Weight increment after 7 weeks in GW(2 mg/kg), GW + OlGly and GW + OlAla treated mice. **F** Weight increment after 7 weeks of HFD-O in the vehicle and GW (2 mg/kg) treated mice; *n* = 6 mice per group, *p < 0.05, Unpaired *t*-test with Welch’s correction. STD line indicates the mean of the weight increment in mice under standard diet (STD) (**G**) Cumulative food intake (g) in mice under chow diet and HFD-O; *n* = 12 chow, *n* = 6 HFD-O ***p < 0.001, Unpaired *t*-test with Welch’s correction. **H** Cumulative energy intake (Kcal) in mice under chow diet and HFD-O; *n* = 12 chow, *n* = 6 HFD-O ****p < 0.0001, Unpaired *t*-test with Welch’s correction.

To test the hypothesis that OlGly and OlAla (50 mg/kg), by virtue of their inhibition of the CPP induced by HPF, could reduce weight gain associated with chronic consumption of an HPF, we treated two groups of mice fed with HFD-L. Both molecules reduced weight gain and food intake in these mice (Fig. 3A–D), while no difference in the weight increment was observed as compared to GW alone when mice were treated with GW, GW + OlGly or GW + OlAla (Fig. 3E). Interestingly, mice fed with an isocaloric HFD diet containing olive oil (HFD-O) instead of lard behaved as chow-vehicle mice after 7 weeks of treatment as far as weight gain (Fig. 3F). GW administration in mice fed with HFD-O, but not HFD-L, induced a significant increment in weight gain, supporting the possible existence of endogenous PPARα activators, possibly derived from olive oil, in the control of weight gain (Fig. 3F). The analysis of cumulative food intake revealed that the HFD-O mice ate overall slightly less than the mice fed with the chow diet (Fig. 3G), although their caloric intake was higher (Fig. 3H).

### OlGly and OlAla reduce the action potential discharge of putative VTA DA neurons

**Fig. 4:**
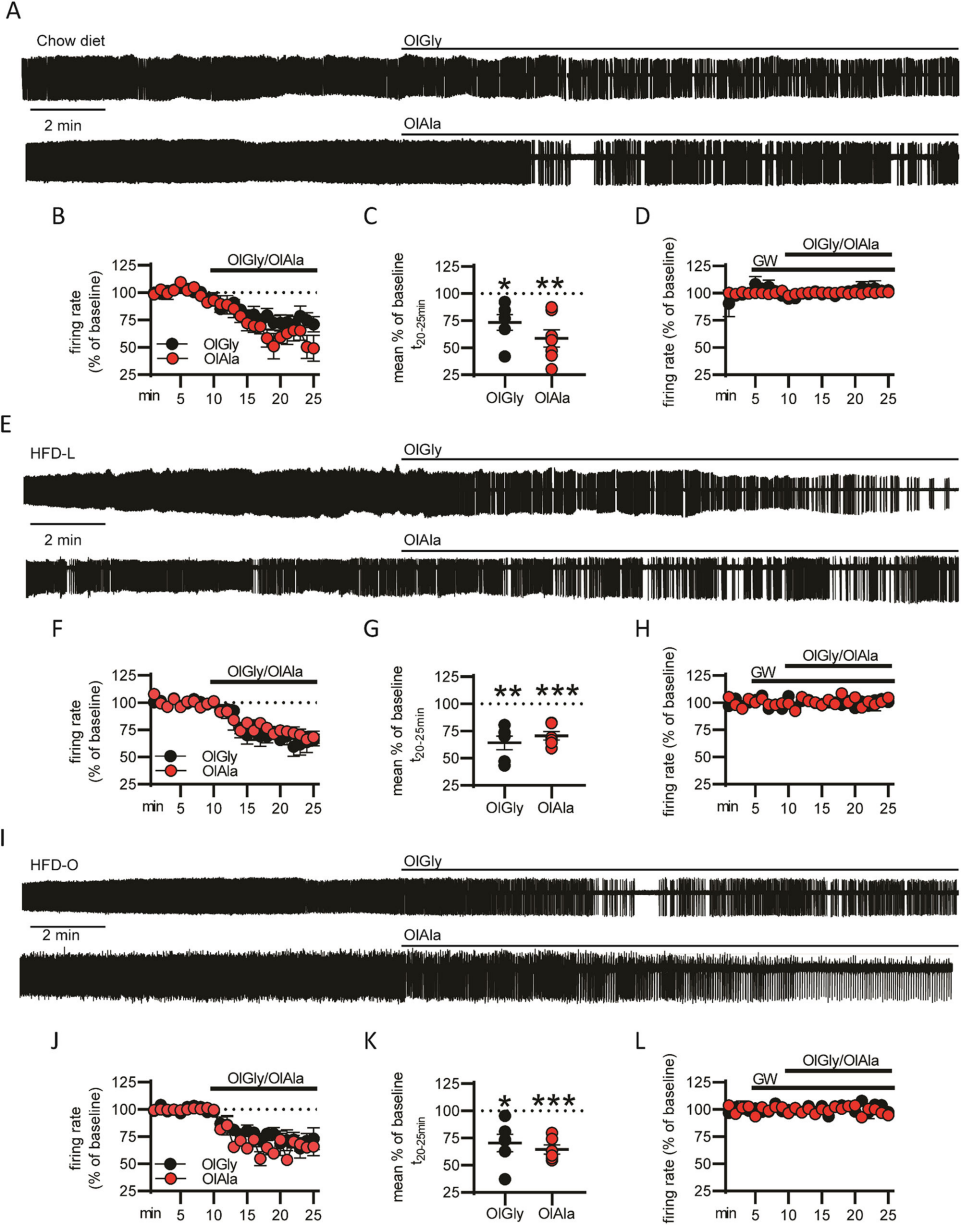
OlGly and OlAla reduce the firing rate of putative DA neurons in the VTA of mice. **A** Representative trace from a putative DA neuron before and after OlGly and OlAla (10 μM) treatment in mice under a chow diet regime. **B** Percentage of firing rate changes after addition of OlGly or OlAla in comparison to the baseline; statistical significance was calculated comparing the average of the firing rate in the last 5 min, **C** Plot of the mean firing rate in percentage of baseline calculated in the interval t_20-25_. *n* = 6 neurons from *n* = 3 mice per group. *p < 0.05 and **p < 0.01 One sample *T*-test. **D** Percentage of variation of the firing rate in slices pretreated with GW6471 (100 nM) with respect to the baseline after the addition of OlGly or OlAla. *N* = 3 and 4 neurons, respectively, per group from 3 mice. **E** Representative trace from a putative DA neuron before and after OlGly and OlAla treatment in mice under a HFD-L. **F** Percentage of firing rate changes after addition of OlGly or OlAla in comparison to the baseline; statistical significance was calculated comparing the average of the firing rate in the last 5 min. **G** Plot of the mean firing rate in percentage of baseline calculated in the interval t_20-25_. *n* = 6 neurons from *n* = 3 mice per group. **p < 0.01, ***p < 0.001, One sample *T*-test. **H** Percentage of variation of the firing rate in slices pretreated with GW6471 (100 nM) with respect to the baseline after the addition of OlGly or OlAla. *N* = 3 neurons, respectively, per group from 3 mice each. **I** Representative trace from a putative DA neuron before and after OlGly and OlAla treatment in mice under a HFD-O. **J** Percentage of firing rate changes after addition of OlGly or OlAla in comparison to the baseline; statistical significance was calculated comparing the average of the firing rate in the last 5 min. **K** Plot of the mean firing rate in percentage of baseline calculated in the interval t_20-25_. *n* = 6 neurons from *n* = 3 mice per group. *p < 0.05, ***p < 0.001, One sample *T*-test. **L** Percentage of variation of the firing rate in slices pretreated with GW6471 vs the baseline after the addition of OlGly or OlAla. *N* = 3, respectively, per group from 3 mice.

HPF significantly activates the VTA and strengthens the dopamine reward circuitry^3,4^. To analyze the effect of OlGly and OlAla on putative VTA DA neurons^14^, loose cell-attached recording was performed in brain slices and only neurons with electrophysiological and anatomical features specific of DA neurons were selected for the study (Supplementary Fig. 1A–C)^14,22^. OlGly and OlAla (10 μM) were added to the recording chamber after 10 min of baseline measurement. Both molecules reduced the firing rate of putative DA neurons of mice under an *ad libitum* chow diet (Fig. 4A–C). However, pretreatment with GW (100 nM) inhibited the effect of both OlGly and OlAla on VTA DA neuron firing rate (Fig. 4D). Very similar results were obtained in slices from mice under HFD-L (Fig. 4E–H) or HFD-O (Fig. 4I–L).

These results point to PPARα activation as a possible mechanism of action of OlGly and OlAla in the control of the addictive effects of HPF through the reduction of VTA DA neuron firing rate^23^.

### The concentrations of OlGly and OlAla in brain areas involved in reward are increased in HFD-O mice as compared to HFD-L and/or chow mice

We, therefore, next measured by IT-TOF MS-MS coupled to liquid chromatography^9,12^ the tissue concentrations of OlGly and OlAla in various brain regions involved in reward (VTA, accumbens, insula and hypothalamus) from mice fed with chow, HFD-O or HFD-L for 7 weeks. In chow mice, OlGly and OlAla were detected only in the insula and VTA. In these two regions, OlGly levels tended to be higher in the HFD-O group as compared to the other two groups, while, in the accumbens and, particularly, the hypothalamus, the HFD-O diet led to the appearance of detectable or even quite high concentrations of this mediator (Supplementary Fig. 2). Although, in HFD-O mice, OlAla levels in the VTA were not higher than the in chow mice, nor did they become detectable in the hypothalamus and accumbens, a trend towards an increase was found in the insula. Additionally, HFD-L mice exhibited significantly reduced or undetectable concentrations of this compound in the insula and VTA, as well as those of OlGly in all brain regions, as compared to chow and/or HFD-O mice (Supplementary Fig. 2). The levels of the well-established endogenous PPARα agonist, *N*-oleoylethanolamine (OEA)^24^, were at least one order of magnitude higher than those of OlGly or OlAla in all brain regions and were not significantly altered by the HFD-O diet in any of the four regions analysed, and in fact tended to be lower in the VTA, accumbens and insula of HFD-O mice (Supplementary Fig. 2).

In order to understand if endogenous intestinal signaling of the two mediators is increased following the intra-peritoneal (i.p.) administration of the exogenous compounds, we analysed OlGly and OlAla concentrations in the large intestine of chow and HFD-L mice w/o treatment with the exogenous compounds, and in HFD-O mice as a comparison. We found that: (1) the administration of the two compounds did not significantly alter their levels in chow mice, but it strongly increased them in HFD-L mice, possibly due to the fact that the intestine expresses high levels of amidases for endocannabinoid-like mediators, such as fatty acid amide hydrolase (FAAH), which can be inhibited by fats^25^, and (2) the HFD-O diet led to levels that were much higher than those of HFD-L and, in the case of OlGly, comparable to those obtained with the exogenous administration of the two compounds in HFD-L mice (Supplementary Fig. 3).

### OlGly, OlAla and olive oil affect intestinal microbiota composition in chow or HFD mice

**Fig. 5:**
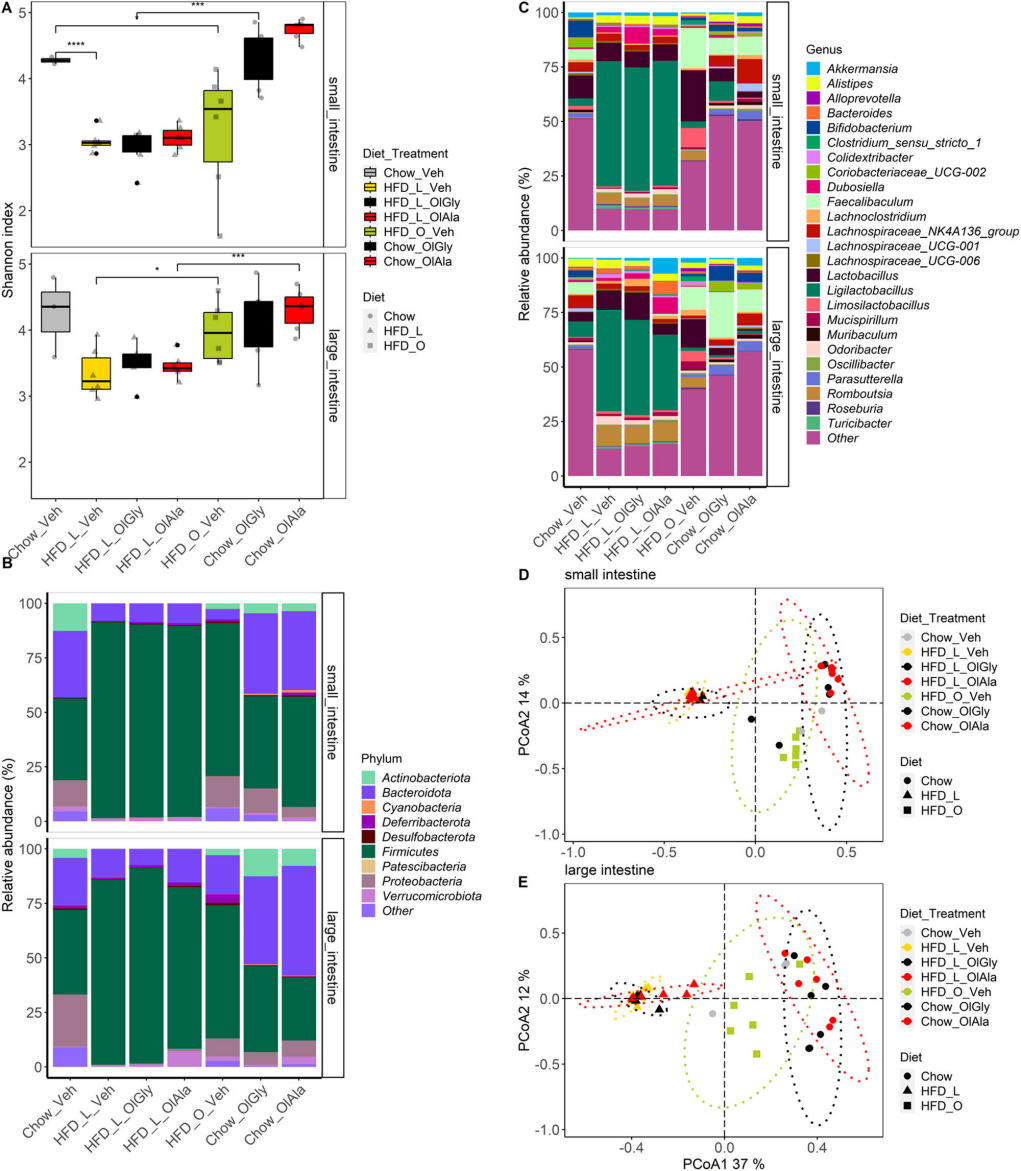
Olive oil diet counteracts the decrease of microbiota diversity observed under HFD-L and displays a distinct microbiota composition at the interface between HFD-L and chow diets. **A** Shannon alpha diversity displayed for each diet and treatment combined condition. *T*-test: *p < 0.05, **p < 0.01, ***p < 0.001, ****p < 0.001. *P-*values were corrected for multiple comparisons using the Bonferoni method. Proportion of bacterial (**B**) phyla, (**C**) genera in each diet and treatment combined condition. Only the top 25 genera are shown in the figure. **D**, **E** PCoA of bacterial genera based on Jaccard dissimilarity in the small (**D**) and large (**E**) intestine. The ellipses were drawn for each Diet-Treatment condition.

**Fig. 6:**
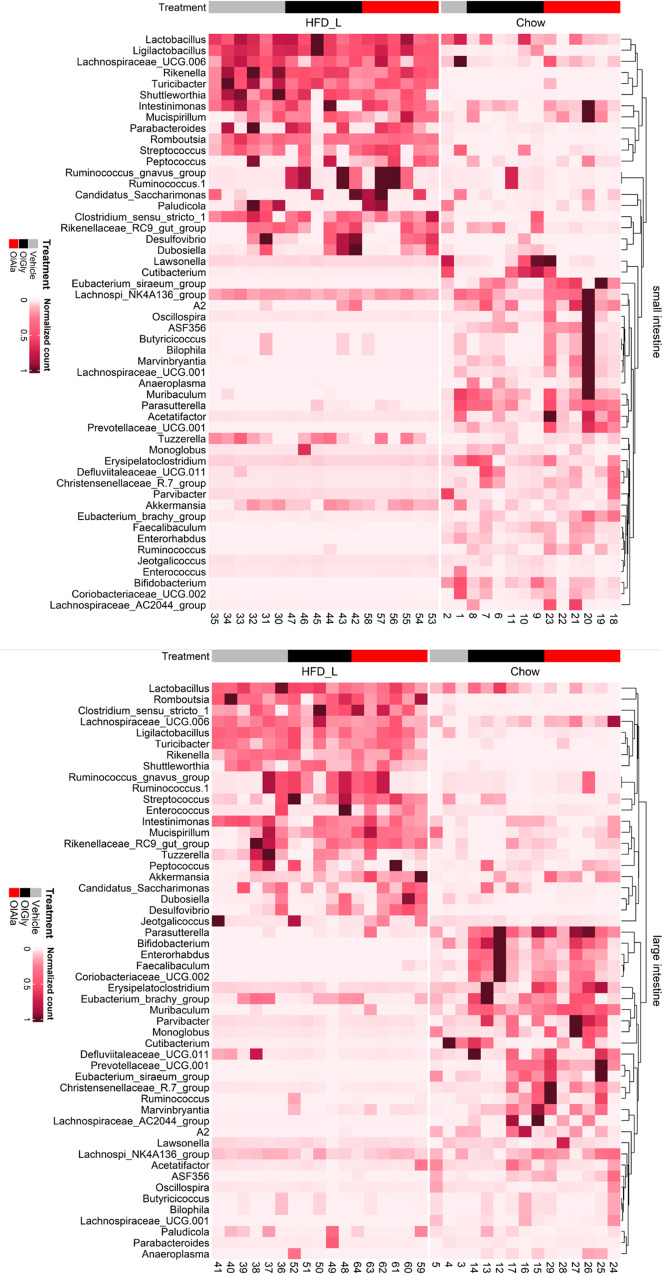
Diet is the main driver of the overall microbial signature shift across gut regions regardless of the treatment. The heatmaps display the DESeq normalized counts of genera that are significantly shifted between diets chow and HFD-L, and/or treatments using a Wald Test. Columns are reordered according to an Unweighted Pair-Grouped Method using arithmetic Averages (UPGMA) clustering dendrogram at the genus level for each gut region.

Given the recently suggested important role of the gut microbiota in food addiction^15,26,27^, we next investigated the effect of OlGly and OlAla administration on the relative taxonomic composition of the small and large intestinal microbiota in mice fed with either chow or HFD-L for 7 weeks and compared it to the effect of HFD-O. First, we observed that the two mediators did not affect α-diversity in chow mice, a measure of microbial diversity and metabolic “health”, assessed with the Shannon index (Fig. 5A), although a trend towards an increase was found with OlAla in the small intestine. Likewise, neither OlGly nor OlAla counteracted the reduced α-diversity typical of HFD-L obese mice, although again a trend was observed with OlAla in the small intestine. On the other hand, feeding with HFD-O resulted in a significant increase in α-diversity in the large intestine compared to HFD-L mice, resulting in a value similar to that of chow mice, whereas in the small intestine, only a non-statistically significant increase was observed *vs*. the HFD-L diet (Fig. 5A, Supplementary Data 2). Based on the phyla and the 20 most abundant genera, each diet, i.e. chow, HFD-L and HFD-O, displayed distinct microbiota signature compositions, and only few changes seemed to be exerted by OlGly and OlAla in either HFD-L and, particularly, chow-fed mice (Fig. 5B, C). PCoA analysis of bacterial genera based on Jaccard dissimilarity in the small (Fig. 5D) and large intestine (Fig. 5E) confirmed that the diet was the major determinant of differences between groups (*p* ≤ 0.05). The heatmap in Fig. 6 based on the DESeq normalized counts of genera (Supplementary Data 3) that were significantly shifted between diets and/or treatments (*p* ≤ 0.05), obtained using a Wald Test, further confirmed this conclusion.

**Fig. 7:**
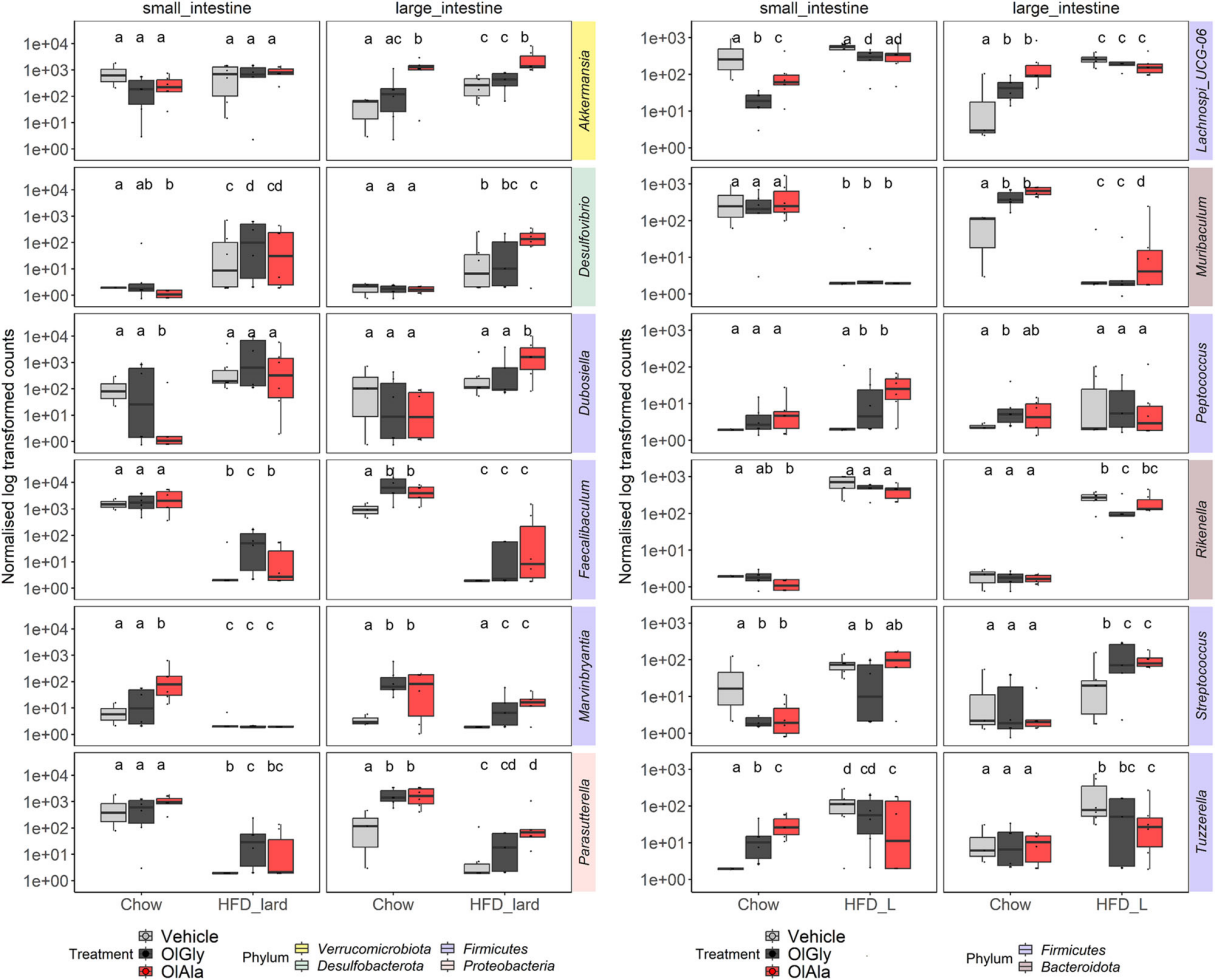
Treatment with OlGly and OlAla induces similar changes in microbiota taxa under HFD-L, but OlAla produces additional changes in the large intestine. Selection of interesting taxa displaying significant changes in normalized genera level abundance between vehicle *vs*. OlGly/OlAla treatments and/or between diets chow *vs*. HFD lard as assessed by DESeq2 analysis in the different gut regions. Pairwise statistical differences per gut region were denoted by letters according to Wald tests. Identical letters indicated no significant differences (*p* > 0.05).

**Fig. 8:**
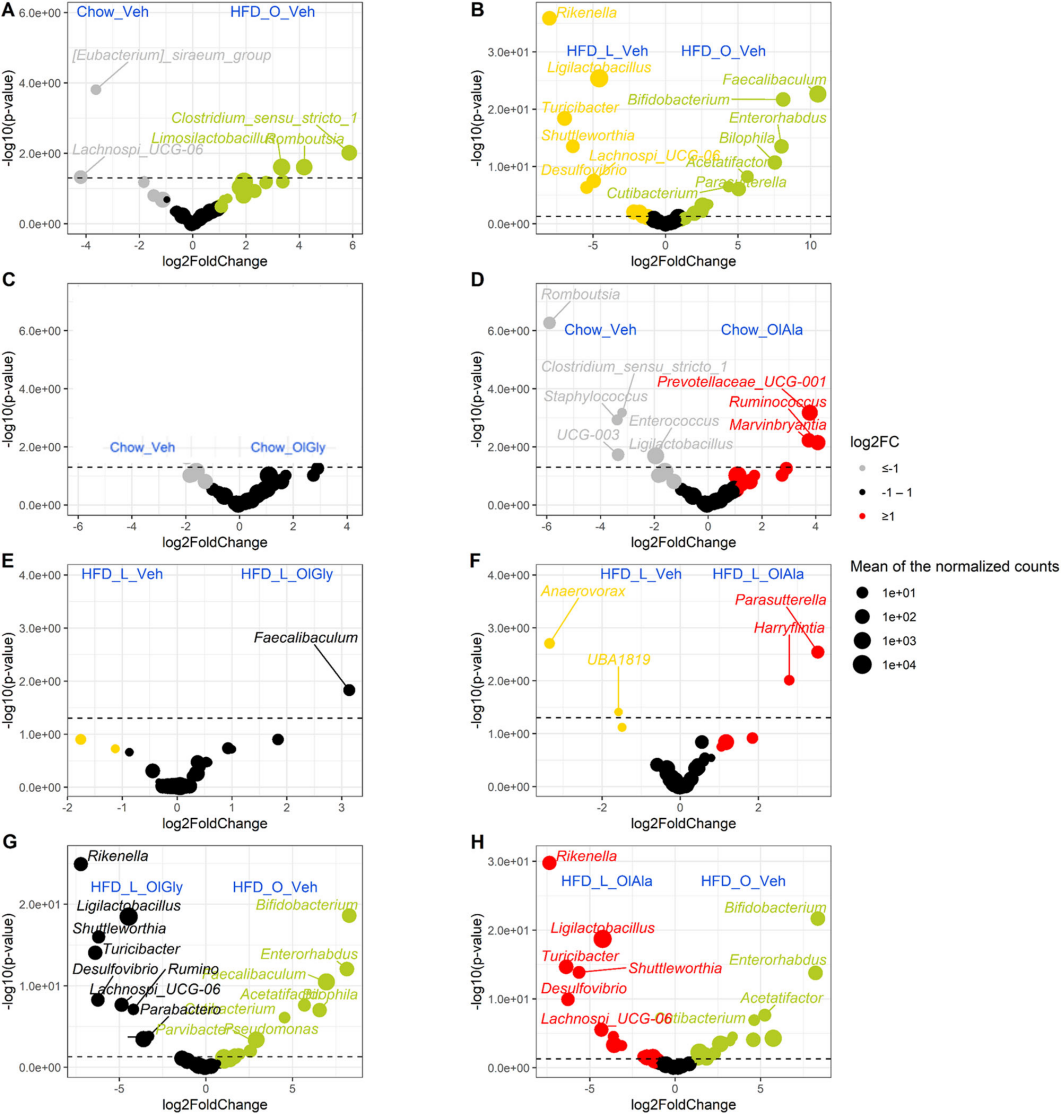
Treatment with HDF-O OlGly and OlAla shows limited microbiota changes with chow diet while it displays increased contrasts with HFD-L. A positive log2 fold-change (FC) indicates a stimulation of the genus under the olive oil diet (HDF-O) (in green) while a negative log2 fold-change indicates a decrease of the genera compared to the chow (in gray) (**A**), as determined by Deseq2 analysis. Additional paired comparisons have been assessed as following: HFD-L-Veh *vs*. HFD-O (**B**), Chow-Veh *vs*. Chow-OlGly (**C**), Chow-Veh *vs*. Chow-OlAla (**D**), HFD-L-Veh *vs*. HFD-L-OlGly (**E**), HFD-L-Veh *vs*. HFD-L-OlAla (**F**), HFD-L-OlGly *vs*. HFD-O (**G**), and HFD-L-OlAla *vs*. HFD-O (**H**). Statistical differences were determined using Wald Tests. The log transformed adjusted *p-value* is displayed on the *y*-axis and the α = 0.05 significance level is indicated by a dashed line.

A more in-depth analysis of the single genera, however, highlighted how OlGly and OlAla did induce changes in microbiota genera under HFD-L in both segments (Fig. 7). These changes were similar between the two compounds, although OlAla produced additional changes in the large intestine (*p* ≤ 0.05) and often produced quantitatively stronger changes (Supplementary Fig. 4, Supplementary Data 4). In particular: (1) *Akkermansia* and *Marvinbryantia* are two well established candidate indicators for persistent success in weight loss^28^. *Akkermansia* was significantly increased by OlAla under chow and HFD-L in the large intestine. *Marvinbryantia*, known to be negatively associated with food addiction in obese women^29^, was significantly increased by both OlGly and OlAla, mostly in the large intestine under both chow and HFD-L (*p* ≤ 0.05) (Fig. 7); (2) *Desulfovibrio*, which also negatively correlates with body weight^29^, and *Dubosiella*, for which contrasting results exist in this sense, but is considered a short chain fatty acid-producing genus and as such would play beneficial actions in HFD-induced obesity and reward^30,31^, tended to be increased, and significantly by OlAla (*p* ≤ 0.05), in chow mice (Fig. 7); (3) *Faecalibaculum* has been associated with obesity^32^, but in the present study its relative abundance was strongly decreased by the HFD-L diet, and OlAla and OlGly tended to partially restore it to levels typical of chow mice in the large intestine and significantly for OlGly in the small intestine (*p* ≤ 0.05, Fig. 7); (4) *Parasutterella*, for which contrasting results also exist, has also been inversely correlated to HFDs^33^, as in the present study, and was again significantly increased by OlGly in the small intestine and OlAla in the large intestine (*p* ≤ 0.05, Fig. 7); (5) *Rikenella* is associated with obesity^34,35^, again as in our study, and tended to be decreased by OlGly/OlAla in the small intestine, and significantly by OlGly in the large intestine (*p* ≤ 0.05, Fig. 7); (6) the increase of *Peptococcus* in the small intestine by the two metabolites can also be considered beneficial^36^ (Fig. 7); (7) *Tuzzerella*, which was significantly increased by HFD-L compared to chow (*p* ≤ 0.05), and is known to be associated with metabolic disorders, tended to be reduced by both molecules in the large intestine and significantly for OlAla in all gut regions (*p* ≤ 0.05)^37^ (Fig. 7); (8) *Streptococcus* and *Lachnospiraceae_UCG006*, two genera that were recently positively associated with food addiction in obese women^29^, were significantly increased by HFD-L mostly in large intestine compared to chow, and were significantly reduced by both OlGly and OlAla in the small intestine of chow mice (Fig. 7); (9) finally, *Muribaculum* is associated with antiobesity effects when increased^38^, and was significantly increased by OlAla in the large intestine under HFD-L and by both compounds under chow (*p* ≤ 0.05, Fig. 7).

The volcano plots of the above mentioned modulations, shown in Fig. 8 (Supplementary Data 5), which group both the small and large intestine, indicate that the HFD-O diet, similar to what described above for OlAla and/or OlGly using single genera comparisons, produced a significant albeit small decrease in the relative abundance of *Lachnospiraceae_UCG006* with respect to chow (*p* ≤ 0.05, Fig. 8A), and the increase of *Parasutterella* and *Faecalibaculum* and the decrease of *Lachnospiraceae_UCG006* and *Rikenella* with respect to the HFD-L diet (Fig. 8B). Volcano plots also confirmed how OlGly and OlAla treatment led to significantly enhanced *Faecalibaculum* or *Parasutterella*, respectively, as compared to HFD-L (*p* ≤ 0.05, Fig. 8E, F), while only OlAla imparted on chow mice significant differences (Fig. 8C, D, *p* ≤ 0.05) that were not all discussed above but could still represent beneficial alterations, i.e the increased abundance of *Prevotella*c*eae-UCG001* and the above-mentioned one of *Marvinbryantia* (Fig. 8F).

In summary, some of the alterations of intestinal microbiota genera induced by OlAla and OlGly, in either chow or, particularly, HFD-L mice, can be interpreted as beneficial against obesity and food addiction and were largely reproduced by HFD-O, which however produced many more changes. This is also exemplified by the volcano plots comparing the intestinal microbiota of HFD-O mice with those of HFD-L treated with either OlAla or OlGly, which showed several differences including a significant stimulation, under HFD-O, of *Bifidobacterium*, known for its health-related properties (Fig. 8G, H). In general, OlAla appeared to produce stronger effects on the gut microbiota (Fig. 7, Fig. 8C–F, Supplementary Fig. 4), possibly due to the fact that this molecule is more metabolically stable to enzymatic hydrolysis than OlGly^8^.

## Discussion

In the present study, we aimed at assessing: (1) whether OlGly and OlAla, two oleic acid-derived mediators belonging to the large family of the eCBome and previously found to relieve either nicotine or alcohol preference or opiate dependence symptoms, or both (refs. ^8,9,12,39,40^), are capable of interfering with food addiction in mice, and consequently with HFD-induced obesity, and through what neural and peripheral mechanisms, and (2) to what extent these putative properties could explain the protective effects of olive oil against HFD-induced obesity. In order to answer these questions, and also to assess to what extent the intestinal microbiome is involved in the putative protective effects of OlGly, OlAla and olive oil, we applied pharmacological, electrophysiological and profiling (targeted lipidomics and metataxonomics) techniques to adult male mice fed with chow or with isocaloric 7-week diets with high fat content (60% of total daily calories) coming from either olive oil (HFD-O) or lard (HFD-L).

We first found that 7 weeks of treatment with 50 mg/kg of OlGly or OlAla did not alter weekly, final or cumulative food intake and body weight. Conversely, both molecules counteracted the CPP associated with a HPF (choco rice cereal), an effect that was not accompanied by inhibition of preference for HPF and, at least for OlGly, seemed to be exerted *via* PPARα activation.

OlAla was previously shown to have a longer-lasting effect (60 min) in rats compared with OlGly due its higher resistance to hydrolysis^8^. It is thus possible that the mere fact that OlAla is more metabolically stable than OlGly renders the effect of the former compound, at least during the CPP test, less amenable to be counteracted by the PPARα antagonist. Additionally, OlAla was suggested to produce a stronger and more prolonged inhibition of FAAH than OlGly, and hence cause indirect activation of PPARα as well as other receptors via elevation of the tissue levels of the endogenous substrates of this enzyme^8^. We may, therefore, hypothesize two effects: one in which OlAla acts directly on PPARα, and a second one in which inhibition of FAAH, and subsequent indirect activation of more than one receptor by its endogenous substrates, plays a role. It is possible that GW6471, at the dose tested in this study, is unable to counteract the effect of OlAla because this compound, in the CPP test at least, is acting predominantly via the second, FAAH-mediated mechanism. Previous studies in rodents have shown that inhibition of FAAH can indeed impair the reward-related effects of nicotine^41^.

Regarding the apparent discrepancy between the absence of effect on HFP preference and the inhibition of CPP by OlGly and OlAla, this may be explained by two hypotheses. Food preference is based on food taste and metabolic/caloric needs. In contrast, the development and maintenance of CPP is based on associative learning between the unconditioned stimulus (US) and the context, which becomes the conditioned stimulus (CS) after pairing with the US. The simplest explanation for our results is that the two compounds block the formation of US-SC conditioning. However, given the current literature using different paradigms and showing that OlGly and OlAla cannot alter taste avoidance learning when administered alone^8,39,40^, this explanation seems unlikely. Another possibility is that, although they do not alter preference for high-calorie foods, OlGly and OlAla do alter the ability of these foods to acquire incentive value, implying that food consumption might be driven by “homeostatic eating” rather than “hedonic eating”^42^ and thus HPF never acquires the properties of an US. Homeostatic eating could develop as a result of daily intake of a HFD, which gradually increases energy requirements while promoting weight gain. In humans, “hedonic eating” (and physiological mechanisms that may mediate it) can be defined as a subjective state, but not as actual "food intake”. In rodents, it has been elegantly shown that the intake of standard chow in food-deprived animals, the motivation to seek HPF, and HPF preference can be independently modulated by different subregions of the nucleus accumbens shell^43^. The results reported in this study suggest the intriguing hypothesis that it is possible to selectively alter these different processes involved in food consumption by pharmacological treatments. Future studies using instrumental paradigms selectively targeting each of these three aspects will be necessary to dissect the precise motivational and consumption mechanisms regulated by the two compounds.

Electrophysiological recordings in brain slices of putative VTA-DA neurons, showed that OlGly and OlAla reduce the firing of these neurons, through the activation of PPARα. It is important to underline the fact that not always there is a direct correlation between the firing rate and the release of dopamine in these neurons (see, as an example, the effect of cocaine on DA neurons^44^). Therefore, the absence of direct data on dopamine release remains a limitation of this study.

OlGly and OlAla chronic treatment reduced cumulative body weight gain and food intake in mice fed on HFD-L, an effect that again was not seen when mice were co-treated with the PPARα antagonist. Collectively, these results report, to the best of our knowledge, antiobesity and anti-food addictive properties for OlGLy and OlAla and, more generally, their efficacy in the treatment of addictive behaviors consequent to a long consumption of HPF.

It is important to note that OlGly and OlAla may act also via peripheral PPARα, which is highly expressed in duodenal and jejunal enterocytes^45,46^. Indeed, activating PPARα receptors on vagal sensory afferences were shown to reduce fat intake through both epithelial cell-mediated and hypothalamic^47,48^ and striatal^49^ feeding circuits.

Interestingly, the HFD-O diet induced an increase in weekly or cumulative body weight after 7 weeks very similar to chow, despite it being isocaloric with the HFD-L diet, and, accordingly, it slightly decreased cumulative food intake. The analysis of food intake underlines a decrease in these parameters, which could be explained also by a decrease in palatability. On the other hand, it has been reported that in mice the liking of olive oil decreases with concentration^50^. In another study using BALB/c mice, it was reported that mice prefer 16- and 18-carbon unsaturated long-chain fatty acids such as oleic acid at low concentrations (1%)^51^. Our HFD-O diet contains 18% oleic acid, the maximum amount of olive oil that can be used for a solid diet. Additionally, although we did not measure energy expenditure changes induced by the diets, the HFD-O may have produced a positive effect on this parameter. Indeed, olive oil has been previously shown to increase energy expenditure in both rodents and humans^52,53^.

At any rate, we observed that the effects of HFD-O on both food intake and body weight gain were undistinguishable from those observed with an equicaloric HFD-L diet in the presence of either OlGly or OlAla, unless this diet was administered in the presence of the PPARα antagonist. These findings raise the possibility that the HFD-O diet might act, in part, by providing dietary oleic acid necessary for the formation of endogenous PPARα agonists, such as OlGly and OlAla, in brain areas involved in reward control. Indeed, by using a targeted lipidomics approach, we found that the concentrations of OlGly were, or tended to be, increased in all four brain areas investigated, including the VTA, shown above to respond to this compound with a reduction in putative DA neuron firing in a PPARα–mediated manner. This possibly indicates that the HFD-O, unlike the HFD-L, might counteract the rewarding-associative properties of the excess fat present therein partly by increasing OlGly levels in the brain and hence indirectly activating PPARα in brain regions deputed to contribute to food addiction. Interestingly, the HFD-L diet decreased, instead, the levels of both OlGly and OlAla in the four analysed brain areas as compared to the HFD-O and/or chow diet, again suggesting that HFDs poor in oleic acid and rich in saturated fatty acids may owe their hedonic and food intake-inducing properties in part to their inability to induce the formation of endogenous anti-food addiction OlAla and OlGly. This mechanism might be specific for OlAla and OlGly, since the levels of the best known endogenous, oleic acid-derived, PPARα agonist, OEA, did not increase, following HFD-O, in the four brain areas analysed, in agreement with the proposed selective peripheral role (at the level of the small intestine) of OEA anorexigenic actions^54^. However, to investigate if the mechanism of action of OlGly and OlAla, and their commonalities with that of the HFD-O diet, included also gut-mediated mechanisms, we examined their effects on the intestinal microbiota in both mice fed with chow and HFD-L. We found that the two compounds, unlike the HFD-O diet, did not significantly affect gut microbial diversity. However, OlGly and, particularly, OlAla did produce, in both the small and large intestine, several taxonomic shifts that could be reconciled with their anti-obesity and anti-food addiction actions, particularly in view of recent findings that have suggested a strong association between this latter pathological propensity and the composition of the gut microbiota^15,26,27,55–57^. In particular, the increase and decrease, respectively, of three taxa that are known to be negatively (*Akkermansia*) and positively (*Streptococcus* and *Lachnospiraceae*) associated with food addiction^29^, by the two compounds may have played a role in their anti-addictive actions. However, the HFD-O diet, beyond its positive effect on α-diversity, which has been recently associated with low food addiction and body weight in women^58^, seemed to produce stronger, and potentially beneficial, eubiotic effects than OlAla and, particularly, OlGly. As an example, HFD-O significantly increased the relative abundance of *Bifidobacterium* compared to HFD-L-OlGly/OlAla. Accordingly, our clustering analysis clearly indicates that the diet, much more than the treatment with OlGly or OlAla, is the strongest determinant of gut microbiota compositional changes. This was not surprising for at least three reasons: (1) the HFD-O diet, and olive oil in particular, contain several components in addition to triglyceride-esterified oleic acid, such as polyphenols, which are known to affect gut microbiota composition^59^; (2) olive oil could also act as a precursor of mediators other than OlGly and OlAla, and in tissues beyond the brain, such as intestinal OEA, which is known to produce beneficial effects on gut microbiota composition along with reduction of food intake and fat adsorption by the intestine^60,61^—this could also explain the slight anorexiant effects of the HFD-O, which was not observed with OlGly and OlAla; and (3) unlike the HFD-O diet, OlGly and OlAla had to be administered i.p. and not *per os*, and this could have attenuated their effects on the gut microbiota—indeed, in agreement with their stronger effects on the HFD-L mouse gut microbiota, we found that i.p. administration of OlGly and OlAla resulted in a strong increase of the intestinal concentrations of the two compounds (in the case of OlGly comparable to that obtained with HFD-O) only when administered together with the HFD-L. This finding is in agreement with previous reports showing that intestinal fat limits the inactivation by endogenous amidases of long chain fatty acid amides^25^. Nevertheless, OlGly and, particularly, OlAla, on the one hand, and olive oil, on the other hand, did produce some common alterations in the gut microbiome, such as the small decrease in *Lachnospiraceae_UCG006* with respect to chow, and the increase of *Parasutterella* and *Faecalibaculum* and the decrease of *Lachnospiraceae_UCG006* and *Rikenella* with respect to the HFD-L diet. Due to the above-mentioned proposed involvement of these taxa in food addiction or obesity, these changes could have partly contributed to the similar effects of the three treatments on food intake and body weight gain under high-fat feeding.

In conclusion, we have shown here that two eCBome mediators, OlGly and OlAla, inhibit conditioned preference for HPF, reduce the putative DA neuron firing in the VTA of brain slices, and reduce body weight and food intake in mice under an HFD rich in lard (i.e. mostly saturated fats). These effects appear to be mostly antagonised by PPARα blockade, which instead increased body weight accumulation in mice under an HFD-O (i.e. richer in monosaturated fats, and oleic acid in particular), thus allowing us to suggest that the effect of the latter diet might be partly mediated by OlGly and OlAla, whose brain and intestinal levels were indeed modulated by HFD-O and HFD-L in opposing manners. Like with the HFD-O diet, the effects of OlAla and OlGly were also accompanied, in both chow-lean and HFD-L-obese mice, by potentially beneficial changes in the intestinal microbiota composition, although this study was not designed to assess the causal role of the gut microbiome in the actions of the two compounds. A parsimonious interpretation of our data is that OlGly and OlAla produce effects on food intake and body weight gain in mice under an HFD similar to those of olive oil, via partially overlapping mechanisms (i.e PPARα activation and intestinal microbiota modulation). However, it is important to note that our data do not allow us to exclude that other previously reported peripheral or central PPARα-mediated mechanisms, proposed to mediate for example the action of the other oleic acid-derived endocannabinoid-like compound, OEA, such as the inhibition of intestinal fat adsorption, adipogenesis and insulin resistance, and the enhancement of satiety and energy expenditure, may mediate the anti-obesity effects of OlAla and OlGly^62–65^. At any rate, the results of this study warrant further investigation on the capability of olive oil-rich diets, such as the Mediterranean diet, to produce beneficial effects via actions on food reward of oleic acid-derived eCBome mediators.

## Methods

### Animals and drugs

Experiments were performed following the European Union animal welfare guidelines [European Communities Council Directive of September 22, 2010 (2010/63/EU)] and the Italian Decree n.26/2014, authorization n. 152/2020-PR and n. 2013/0040360. The experiments were performed on 8−13-week-old mice. All the mice were housed in controlled temperature (20−23 °C) and humidity conditions (55 ± 5%) and fed ad libitum with a VFR1 standard diet (Sniff). For the HFD experimental group, C57Bl/6j were fed with an HFD-L where 60% kcal derived from Lard (233HFD-D12492, SAFE-Lab; 31.7%, 3.3% soya oil) for 8 weeks starting from 1 month of life or with an HFD-O where 17% of the total fat derived from olive oil (D12492, SAFE-Lab; lard 14.7%, 17% olive oil, 3.3% soya oil). The fatty acid composition of the two HFD is reported in Supplementary Data 6. Mice were housed one per cage to evaluate food consumption and body weight. A known amount of pellet per cage was positioned and the weight after a week was registered. The drug concentrations used for all the experiments were as follows OlGly and OlAla (50 mg/Kg in vivo i.p.; 10 μM in vitro); GW6471 (2 mg/Kg in vivo i.p.; 100 nM in vitro).

### Conditioned place preference

The experimental design is reported in Supplementary Fig. 1A. Animals were recorded using a video camera (PANASONIC WV‐BP330) connected to a video‐tracking system (ANY‐MAZE 7.08, Stoelting, USA). CPP was carried in a plastic apparatus formed by two compartments of the same size (20 cm length, 10 cm width, 15 cm height)^66^. One compartment had a white floor and the walls covered by black stripes and the other was white. During the conditioning session, the compartments remained closed by a removable door. Before starting the experiment mice were free to explore the two compartments for a 30-minutes period and behavior was monitored to calculate the time spent in each compartment to evaluate if mice showed bias (Fig. 2). We did not observe any preference for one of the two compartments.

During the first 7 days of housing, C57Bl6j mice at 2 months of age were injected on alternate days. OlGly and OlAla were injected 15–30 minutes before starting the experiment^12^. GW6471 was administrated 30 minutes before the administration of OlGly or OlAla^12^. From the 8th to the 11th day, mice were injected every day, according to the protocol depicted in Fig. 2A. From the 5th day of the experiment, the HFP (choco rice cereals) was introduced in the diet of each experimental group for familiarization. On the 6th day, mice received limited access to food (HPF+standard pellet) to induce ∼10–15% weight loss and to increase the mice’s motivation to eat during the test. No differences were observed in the body weight between the experimental groups during the different experimental phases (Fig. 2C). On the 8th, 9th, and 10th day mice were conditioned into the CPP setup by performing two sessions per day (Fig. 2A, B). In particular, on the 8th day, mice were confined 15 min in the room containing chow food and 15 min in the other room containing HPF. The distribution of the chow or HPF in the white room or the room with black stripes (Fig. 2B) was equally subdivided and the mice were randomly assigned to one of the two conditions. On the day 9th and 10th, mice were subjected to a double session of conditioning for 30 minutes in the morning and 30 minutes in the afternoon. During these sessions, the connecting door between the room containing the HPF and that containing chow was opened allowing the mice to freely move in the apparatus and freely choose what to eat among the two types of food. On day 11th (**CPP**_**1**_, Fig. 2A) CPP was tested under an extinction protocol: mice were free to move between the two rooms for a total duration of 30 minutes, no food was administrated during this session and the time spent in the two rooms was calculated.

### Electrophysiology

Preparation of VTA slices: C57BL/6 J 8–9 weeks old of both sexes were used. After anesthesia with an overdose of isoflurane, the mice were decapitated, and the brains were quickly removed. Coronal slices (200 μm) containing the VTA were cut using a Leica VT1000 S Vibrating blade microtome at 3–5 °C in a solution containing (in mM): 87 NaCl, 25 NaHCO_3_, 2.5 KCl, 0.5 CaCl_2_, 7 MgCl_2_, 10 glucose, 75 sucrose, and saturated with 95% O2 and 5% CO2. After cutting, we let the slices recover at least 30 min at 35 °C and for 10 min at room temperature in artificial cerebrospinal fluid (ACSF) containing in mM: 125 NaCl, 25 NaHCO_3_, 10 glucose, 2.5 KCl, 1.25 NaH_2_PO_4_, 2 CaCl_2_, and 1 MgCl_2_ (bubbled with 95% O_2_–5% CO_2_).

Coronal slices were transferred to a recording chamber and continually perfused with gassed ACSF. The recording was performed with a Multiclamp 700B/Digidata1440A system in loose cell-attached configuration. The extracellular recording was performed from visualized putative DA neurons using a Leica DM6000 FS microscope equipped with a WAT-902H Ultimate camera. Recording pipettes pulled using a Sutter P-1000 puller had a resistance of 3–7 MΩ when filled with ACSF. Spontaneous action potentials were detected at 31 °C in voltage clamp (0 mV) lowering the pipette on the neuronal surface and applying a small suction until the series resistance reached a 35–50 MΩ resistance. Putative DA cells were recognized using the following criteria: (a) the anatomic localization and dimension of the cells, (b) the firing rate frequency between 1 and 5 Hz, (c) the action potential duration over 1ms^14,22^ (Supplementary Fig. 1).

 To stabilize the recording configuration, the recording was started 5 min after that the cell-attached configuration was established. One cell for each slice was recorded.

Data were acquired with pClamp 10.4 software (Molecular Devices) and analysed offline with Clampfit 11.1 (Molecular Device), Excel, and GraphPad Prism 8.0.2 (GraphPad Software, USA).

To analyse the variation of the firing rate we plotted the variation in the percentage of the firing rate with respect to the baseline of 10 min calculated as 100%.

### LC/MS-MS analysis

Brain and intestinal tissues were frozen in liquid nitrogen immediately after dissection, which took place within 5 min from sacrifice. Tissues were dounce-homogenized and extracted with chloroform/methanol/Tris-HCl 50 mM pH 7.5 (2:1:1, v/v) containing internal deuterated standards as described in^9^. Then, the lipid extract was dissolved in 100 μl of CH_3_OH and analyzed by either LC-APCI-MS for OEA quantification, which was calculated based on their area ratio with the internal deuterated standard signal areas^67^ and by LC-MS-IT-TOF (Shimadzu Corporation, Kyoto, Japan) for OlGly, OlAla and *N*-oleoylserine identification and quantification, using multiple reaction monitoring (MRM), as reported previously^9^. The chromatograms of the high-resolution [M-H]^-^ values were extracted and used for calibration and quantification. LC analysis was performed in the isocratic mode using a Phenomenex Kintex Polar C18 column (50 × 3 mm, 2.6 μm) and CH_3_OH/water/formic acid (85:15:0.1 by vol.) as the mobile phase with a flow rate of 0.15 ml/min. Identification of OlGly, OlAla and *N*-oleoylserine was carried out using ESI ionization in the negative mode with a nebulizing gas flow of 1.5 ml/min and curved desolvation line temperature of 250 °C. The most dominant product ion for each lipid class (m/z 74 corresponding to glycine and serine loss or [M-H]^−^ m/z 88 corresponding to alanine loss) was selected for MRM.

After having ascertained the presence of OlGly and OlAla in the brain using a high resolution LC/MS-MS method, we proceeded to analyse the intestine of mice treated with OlGly and OlAla, or with the HFD-O, using a more sensitive but low resolution method. These analyses were acquired using a Shimadzu Nexera UHPLC (Kyoto, Japan) coupled online to a triple quadrupole LCMS-8050 (Shimadzu) equipped with an electrospray ionization (ESI) source operating in ESI positive mode. The separation was performed on a Kinetex^TM^ Biphenyl C18 100 × 2.1 mm, 2.6 μm (100Å) (Phenomenex, Bologna, Italy). Flow rate was set to 0.5 mL/min, column oven was set at 45 °C. The mobile phases were respectively: (A) H_2_O plus 1 mM CH_3_COONH_4_ + 0.05% acetic acid *v/v* and (B) ACN: H_2_O 95:5 plus 1 mM CH_3_COONH_4_ + 0.05% acetic acid. The following gradient was used: 0 min, 20%B, 0–5 min 100%B, 5–7 min, 100%B, returning to 20% B in 0.1 MS source parameters: Desolvation Line (DL) 250 °C, Interface: 300 °C, Block heater 350 °C, Nebulizing (N_2_), Drying (N_2_), and Heating (Air) gas pressures: 3, 10, 10 L/min. Each analyte was optimized by infusing standard solution at 0.2 mg/mL, multiple reaction monitoring (MRM) mode. The following parameters were used, d4-OlAla: 358.1000 - 94.1500, Q1 prebias −11 V, CE −17 eV, Q3 prebias −15 V; d2-OlGly: 342.0000 – 78.2000, Q1 prebias −19 V, CE −16 eV, Q3 prebias −30 V, OlGly: 340.0000 – 76.2000, Q1 prebias −19 V, CE −15 eV, Q3 prebias −30 V, OlAla: 354.1000 – 90.1000, Q1 prebias −11 V, CE −17 eV, Q3 prebias −15 V. Dwell time was set to 50 ms for all transitions. Samples were injected in randomized order, a pooled quality control sample was inserted during the batch to monitor system stability over time.

### Gut microbiota analysis

#### DNA extraction and sequencing

The DNeasy 96 PowerSoil Pro QIAcube HT (QIAGEN) was used for fecal DNA extraction. Based on the manufacturer’s protocol, up to 200 mg of frozen stool was aliquoted into PowerBead Pro Tubes (QIAGEN, Hilden, Germany), followed by sample homogenization in lysis buffer, and transfer to the QIAcube HT robot (QIAGEN) for further processing. The V3-V4 hypervariable region of 16S rRNA was amplified using primer pairs F (5′-TCGTCGGCAGCGTCAGATGTGTATAAGAGACAGCCTACGGGNGGCWGCAG- 3′) and R (5′- GTCTCGTGGGCTCGGAGATGTGTATAAGAGACAGGACTACHVGGGTATCTAATCC- 3′). According to the Illumina 16S metagenomic sequencing library protocols (Illumina), the Nextera XT Index kit (Illumina Inc) was applied for the amplicon library preparation. The final PCR products were pooled, followed by paired-end sequencing using the MiSeq 600 cycles Reagent Kit V3 by an Illumina MiSeq System (Illumina, San Diego, CA).

#### Bioinformatics analysis of 16S rRNA gene amplicon data

Demultiplexed raw data files covering all the samples were imported into R studio environment (version 4.2.2, R Core Team)^68^. Amplicon sequence variants (ASVs) were inferred using the DADA2 R package version 1.26.0, applying the recommended workflow^69^. Briefly, sequence reads were first filtered and trimmed with the following parameters: truncQ = 2, truncLen = c(275,195), and maxEE = c(2,2). Filtered reads were denoised using the DADA2 algorithm, which infers the sequencing errors. After removing chimeras, ASVs sequences were subsequently merged and classified using the SILVA database SSU Ref NR 99 release 138 using default parameters^70^. Unassigned taxa and singletons were removed. Sequences detected in <5% of all samples were filtered out with PoolTestR package (0.1.2). Rarefaction curves were constructed to ensure that the samples were sequenced at sufficient depth. To deal with differences in sampling depth, the data were rescaled to proportions for further analysis.

#### Microbiome statistical analysis

Statistical and microbiome analyses were performed in R (version 4.2.2) using ggplot2 (3.4.0) for data visualization; phyloseq (1.22.3) for exploring microbiome profiles^71^; vegan (2.6–4) for computing alpha and beta-diversity indexes^72^; ggpubr (0.5.0) for means statistics comparisons; DESeq2 (1.38.1)^73^ for differential analysis of normalized count data between conditions^74^; and ComplexHeatmap (2.14.0) packages. All statistical tests used were two-sided. All statistical tests were followed by multiple-testing correction using the Benjamini–Hochberg method when testing more than two features. Data distribution was assumed to be normal, but if this was not the case, nonparametric testing or data transformation was applied.

#### Statistics and reproducibility

Statistical analysis was carried out using the GraphPad Prism Software version 8.0.2. Tests were significant when *p* < 0.05. Data are expressed as mean ± SEM. Sample sizes for experiments were determined based on sample sizes used in similar experiments reported previously in the literature. The statistical test used for each comparison is described in the figure legends. The normality distribution of the data was confirmed using D’Agostino-Pearson, Shapiro-Wilk, and Kolmogorov-Smirnov test. When comparing two unpaired groups, data were analyzed by two-tailed Student’s *t*-test (parametric) or a two-tailed Mann-Whitney U test (nonparametric). When two paired groups, data were analyzed by Wilcoxon matched-pairs signed-rank test, two-tailed. When comparing three or more groups, data were analyzed by One-way ANOVA/Bonferroni, Kruskal–Wallis/Dunn’s tests, or 2-way ANOVA with Tukey post hoc were used to analyze data appropriately. Fisher’s LS post hoc tests were run when applicable to identify differences among groups. Pearson’s correlation coefficient was used to analyze the correlation graphs.

### Reporting summary

Further information on research design is available in the Nature Portfolio Reporting Summary linked to this article.

### Supplementary information

Supplementary Figures

Description of Additional Supplementary Files

Supplementary Data 1

Supplementary Data 2–6

Reporting Summary

## Data Availability

Numerical source data are provided in Supplementary Data 1. Raw 16S rRNA gene amplicon sequencing data were deposited under the BioProject accession number PRJNA925185 (NCBI SRA).
