# Supplementary material for: Olive oil-derived endocannabinoid-like mediators inhibit palatable food-induced reward and obesity
Source: Commun Biol. 2023 Sep 21;6:959. doi: 10.1038/s42003-023-05295-y (PMC10514336; doi:10.1038/s42003-023-05295-y)
Supplement: Supplementary file 1 — Supplementary Figures [file 42003_2023_5295_MOESM1_ESM.pdf]

SUPPLEMENTARY DATA

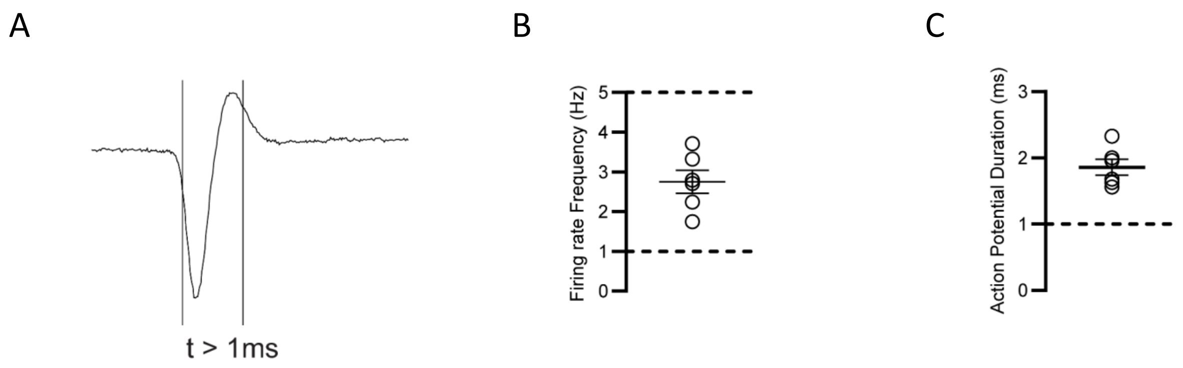

Supplementary Figure 1. Electrophysiological parameters were used to identify putative dopaminergic cells in VTA; (**A-C**) firing rate was between 1 and 5 Hz, and action potential duration was > 1 ms. OlGly and OlAla reduce the firing rate of putative dopaminergic neurons in the VTA via PPARα.

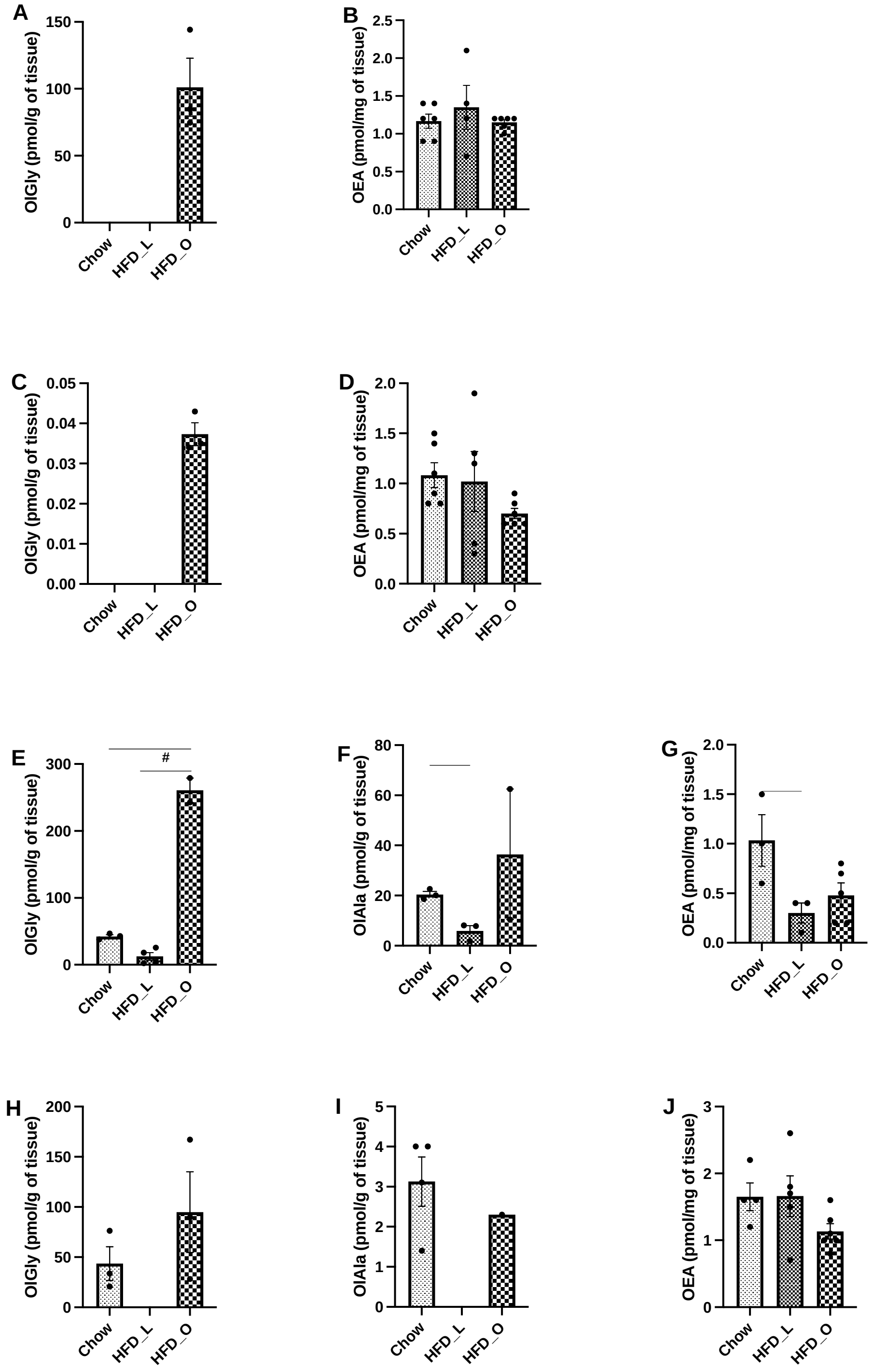

Supplementary figure 2. Oleic acid derivative concentration in brain regions. (**A,B**) OlGly and OEA levels in the hypothalamus. (**C, D**) OlGly and OEA levels in the nucleus accumbens. (**E-G**) OlGly, OlAla and OEA levels in the insula. (**H-J**) OlGly, OlAla and OEA levels in the ventral tegmental area.

* p<0.05, ** p<0.01 Unpaired t-test, Chow vs HFD_L or HFD_O; # p<0.05 HFD_L vs HFD_O

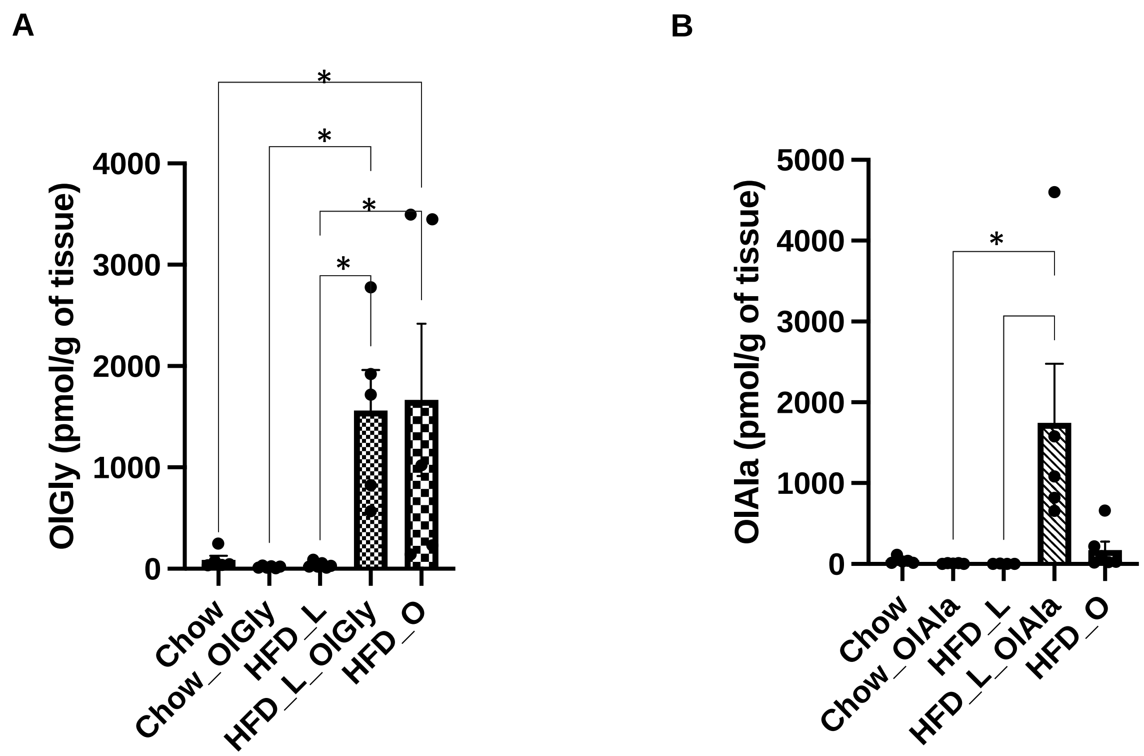

Supplementary figure 3. Oleic acid derivative concentration in the large intestine. (**A**) OlGly levels expressed as pmol/g of tissue.. (**B**) OlAla levels expressed as pmol/g of tissue.
* p<0.05, One-way ANOVA, Tukey post-hoc test

Supplementary Figure 4. (Part 1) Taxa that are significantly modulated between diets and or treatments in each gut region using DESeq analysis.

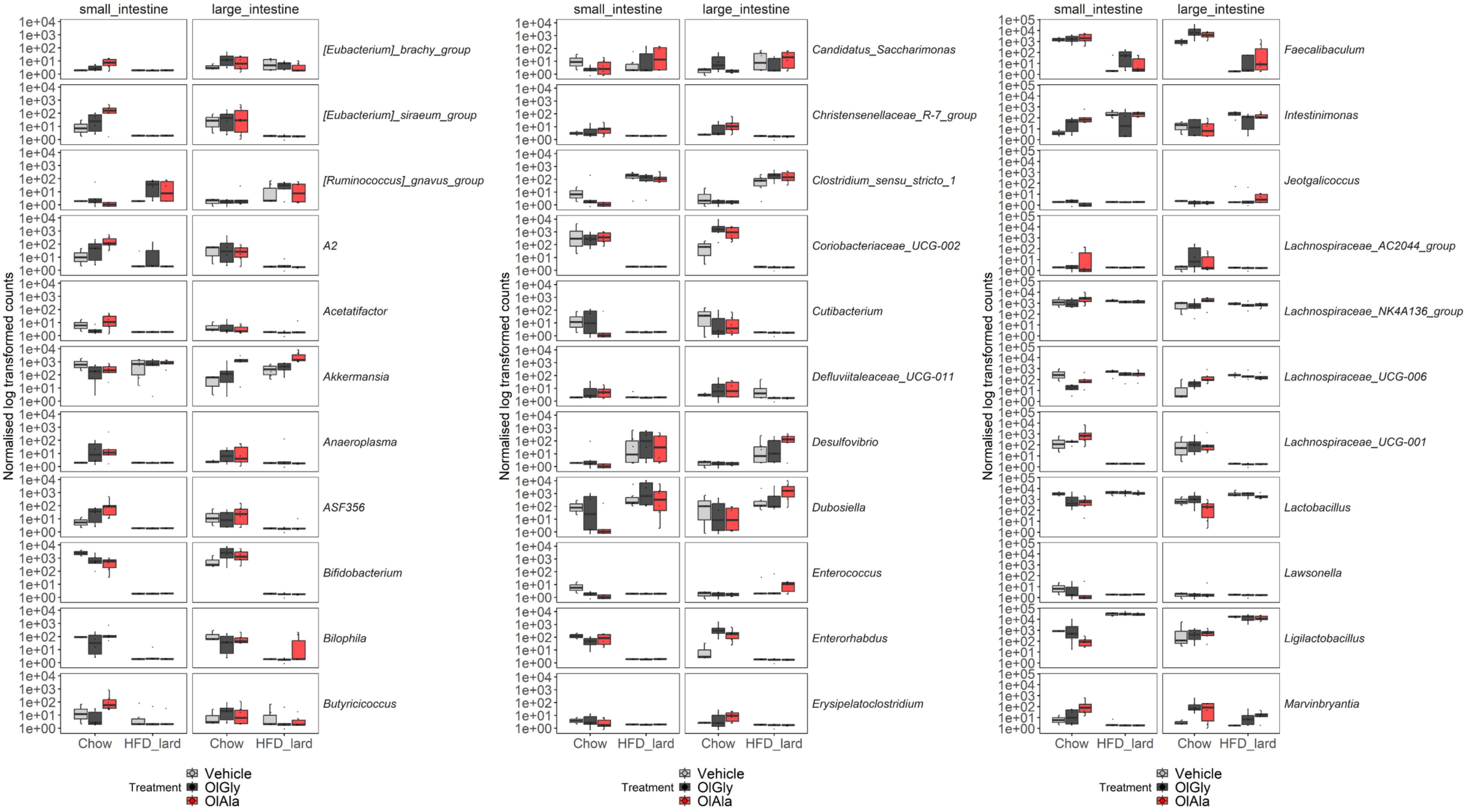

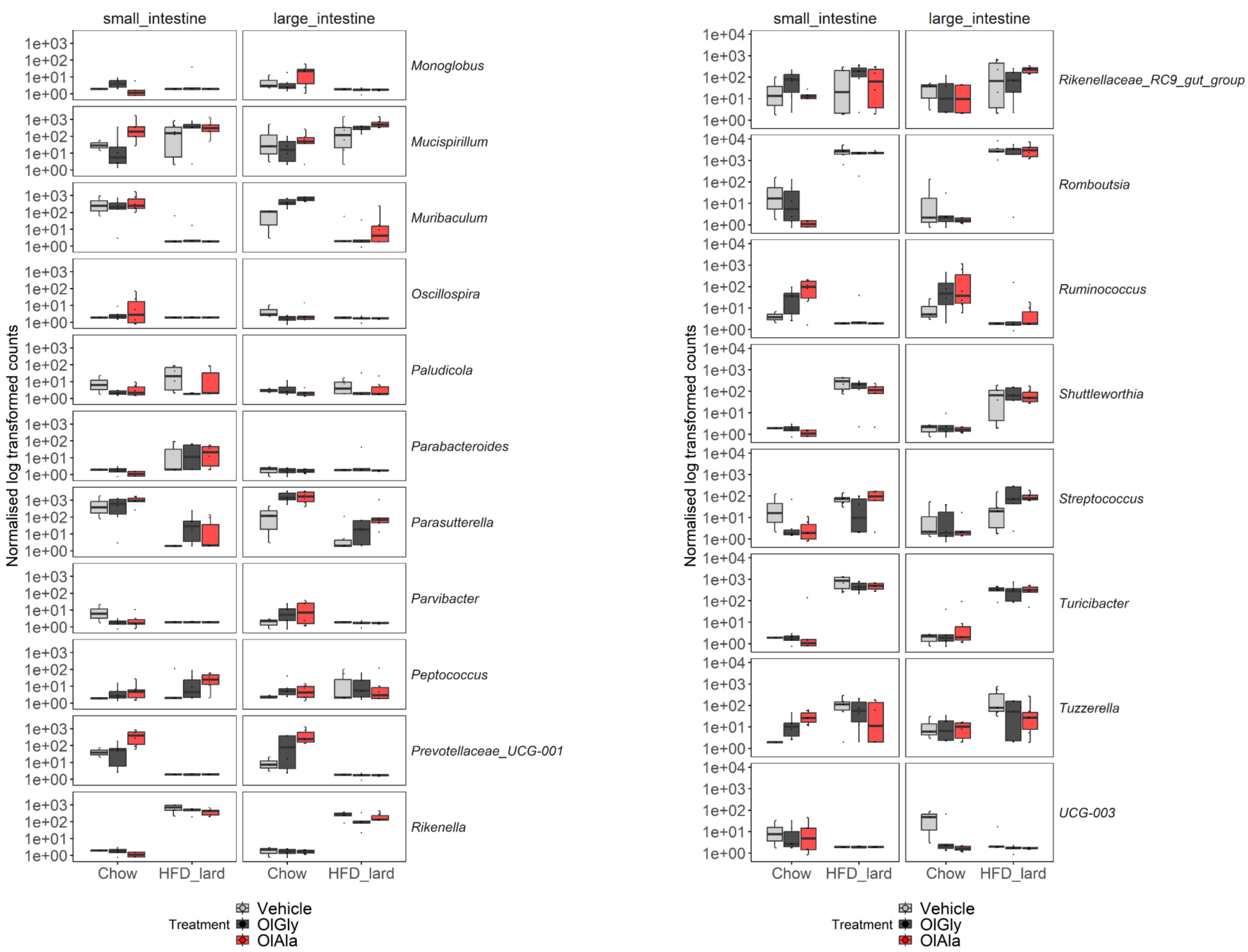

Supplementary Figure 4. (Part 2) Taxa that are significantly modulated between diets and or treatments in each gut region using DESeq analysis.
