# Supplementary material for: Olive oil-derived endocannabinoid-like mediators inhibit palatable food-induced reward and obesity
Source: Commun Biol. 2023 Sep 21;6:959. doi: 10.1038/s42003-023-05295-y (PMC10514336; doi:10.1038/s42003-023-05295-y)
Supplement: Supplementary file 2 — Description of Additional Supplementary Files [file 42003_2023_5295_MOESM2_ESM.pdf]

Description of Additional Supplementary Files

File name: Supplementary Data 1
Description: Source data underlying the graphs and charts presented in the main figures

File name: Supplementary Data 2
Description: Statistics summary of Shannon index using the T test with Bonferonni corrections.

File name: Supplementary Data 3
Description: Rescaled data counts obtained from DESeq and used to build the heatmap of figure 6.

File name: Supplementary Data 4
Description: Statistics summary of taxa counts between treatments and diets in each gut region using the Wald test.

File name: Supplementary Data 5
Description: Statistics summary of log2FoldChange taxa for each volcanoplots using the Wald test.

File name: Supplementary Data 6
Description: Macronutrient, energy and fatty acid compositions of the HFD-L and HFD-O diets.
