# Supplementary material for: Olive oil-derived endocannabinoid-like mediators inhibit palatable food-induced reward and obesity
Source: Commun Biol. 2023 Sep 21;6:959. doi: 10.1038/s42003-023-05295-y (PMC10514336; doi:10.1038/s42003-023-05295-y)
Supplement: Supplementary file 5 — Reporting Summary [file 42003_2023_5295_MOESM5_ESM.pdf]

nature portfolio

Corresponding author(s): Vincenzo Di Marzo, Luigia Cristino

Last updated by author(s): Aug 22, 2023

Reporting Summary

Nature Portfolio wishes to improve the reproducibility of the work that we publish. This form provides structure for consistency and transparency in reporting. For further information on Nature Portfolio policies, see our Editorial Policies and the Editorial Policy Checklist.

Statistics

For all statistical analyses, confirm that the following items are present in the figure legend, table legend, main text, or Methods section.

n/a	Confirmed	
☐	☒	The exact sample size (*n*) for each experimental group/condition, given as a discrete number and unit of measurement
☐	☒	A statement on whether measurements were taken from distinct samples or whether the same sample was measured repeatedly
☐	☒	The statistical test(s) used AND whether they are one- or two-sided<br>*Only common tests should be described solely by name; describe more complex techniques in the Methods section.*
☒	☐	A description of all covariates tested
☐	☒	A description of any assumptions or corrections, such as tests of normality and adjustment for multiple comparisons
☐	☒	A full description of the statistical parameters including central tendency (e.g. means) or other basic estimates (e.g. regression coefficient) AND variation (e.g. standard deviation) or associated estimates of uncertainty (e.g. confidence intervals)
☐	☒	For null hypothesis testing, the test statistic (e.g. *F*, *t*, *r*) with confidence intervals, effect sizes, degrees of freedom and *P* value noted<br>*Give P values as exact values whenever suitable.*
☒	☐	For Bayesian analysis, information on the choice of priors and Markov chain Monte Carlo settings
☒	☐	For hierarchical and complex designs, identification of the appropriate level for tests and full reporting of outcomes
☒	☐	Estimates of effect sizes (e.g. Cohen's *d*, Pearson's *r*), indicating how they were calculated

Our web collection on statistics for biologists contains articles on many of the points above.

Software and code

Policy information about availability of computer code

Data collection	In vitro Electrophysiology: Leica DM6000 FS microscope equipped with a WAT-902H Ultimate camera, MultiClamp 700B and Digidata 1440A (Axon Instruments, Molecular Devices, Sunnyvale, CA, USA) and the pClamp 10.4 software (Axon Instruments), Behaviour: stopwatch (Silva, Sweden), automated behavioral tracking system (Smart v3.0, Panlab Harvard Apparatus), video camera (PANASONIC WV-BP330), video-tracking system (ANY-MAZE 7.08, Stoelting, USA).
Data analysis	In vitro Electrophysiology: Clampfit 11.1.; Behaviour: automated behavioral tracking system (Smart v3.0, Panlab Harvard Apparatus), video-tracking system (ANY-MAZE 7.08, Stoelting, USA). Statistical analysis: GraphPad Prism 8 (GraphPad Software, USA). Bioinformatics analysis of 16S rRNA gene amplicon data: R studio environment (version 4.2.2, R Core Team), using DADA2 package version 1.26.0. Microbiome statistical analysis: R studio environment (version 4.2.2, R Core Team), using packages phyloseq version 1.22.3, vegan version 2-6.4, DESeq2 version 1.38.1 and ComplexHeatmap version 2.14.0.

For manuscripts utilizing custom algorithms or software that are central to the research but not yet described in published literature, software must be made available to editors and reviewers. We strongly encourage code deposition in a community repository (e.g. GitHub). See the Nature Portfolio guidelines for submitting code & software for further information.

Data

Policy information about availability of data

All manuscripts must include a data availability statement. This statement should provide the following information, where applicable:

- Accession codes, unique identifiers, or web links for publicly available datasets
- A description of any restrictions on data availability
- For clinical datasets or third party data, please ensure that the statement adheres to our policy

All data generated or analysed during this study are included in this published article (and its supplementary information files). Raw 16S rRNA gene amplicon sequencing data were deposited under the BioProject accession number PRJNA925185 (NCBI SRA).

Human research participants

Policy information about studies involving human research participants and Sex and Gender in Research.

Reporting on sex and gender	n/a
Population characteristics	n/a
Recruitment	n/a
Ethics oversight	n/a

Note that full information on the approval of the study protocol must also be provided in the manuscript.

Field-specific reporting

Please select the one below that is the best fit for your research. If you are not sure, read the appropriate sections before making your selection.

☒ Life sciences ☐ Behavioural & social sciences ☐ Ecological, evolutionary & environmental sciences

For a reference copy of the document with all sections, see nature.com/documents/nr-reporting-summary-flat.pdf

Life sciences study design

All studies must disclose on these points even when the disclosure is negative.

Sample size	No sample size calculations were performed. The minimal sample-size useful to detect a statistical significattive difference between groups was adopted. Sample size was chosen based on previous experience and standards in the field (De Risi et al, 2021, Nat Comm; Forte et al 2021, Nat Comm)
Data exclusions	Data were not excluded in the analysis
Replication	All experiments were performed at least in Triplicate. For in vitro electrophysiology maximun three slices per mouse were recorded. All attempts of replication were successful and gave similar results.
Randomization	All experiments in this work were performed using animals and each group were selected randomly.
Blinding	For data collection and analysis, the investigators were blinded.

Reporting for specific materials, systems and methods

We require information from authors about some types of materials, experimental systems and methods used in many studies. Here, indicate whether each material, system or method listed is relevant to your study. If you are not sure if a list item applies to your research, read the appropriate section before selecting a response.

Materials & experimental systems

n/a	Involved in the study
☒	☐ Antibodies
☒	☐ Eukaryotic cell lines
☒	☐ Palaeontology and archaeology
☐	☒ Animals and other organisms
☒	☐ Clinical data
☒	☐ Dual use research of concern

Methods

n/a	Involved in the study
☒	☐ ChIP-seq
☒	☐ Flow cytometry
☒	☐ MRI-based neuroimaging

Animals and other research organisms

Policy information about studies involving animals; ARRIVE guidelines recommended for reporting animal research, and Sex and Gender in Research

Laboratory animals	8–13-week-old C57Bl6J male mice
Wild animals	n/a
Reporting on sex	The findings are applicable only to male mice.
Field-collected samples	n/a
Ethics oversight	The study has been performed according to the ARRIVE Guidelines to improve the reporting of bioscience research using laboratory animals. Experiments were performed following the European Union animal welfare guidelines [European Communities Council Directive of September 22, 2010 (2010/63/EU)] and the Italian Decree n.26/2014, authorization n. 152/2020-PR and n. 2013/0040360

Note that full information on the approval of the study protocol must also be provided in the manuscript.
